# Implementing a podiatry prescribing mentoring program in a public health service: a cost-description study

**DOI:** 10.1186/s13047-018-0282-1

**Published:** 2018-07-13

**Authors:** Anna G. Couch, Jonathan Foo, Alicia M. James, Stephen Maloney, Cylie M. Williams

**Affiliations:** 1 Peninsula Health, Community Health, Hastings Rd, Frankston, VIC 3199 Australia; 2 Department of Physiotherapy, Monash University, McMahon’s Rd, Frankston, VIC 3199 Australia

**Keywords:** Podiatry, Prescribing, Endorsement, Scheduled medicines, Cost

## Abstract

**Background:**

In the management of diabetes and high-risk patients, timely treatment with scheduled medicines is critical to prevent severe infections and reduce the risk of lower extremity amputation. However, in Australia, few podiatrists have attained endorsement to prescribe. The aims of this study were to identify the costs associated with developing and implementing a podiatry prescribing mentoring program; and to compare the cost of this program against potential healthcare savings produced.

**Methods:**

This was a cost-description analysis, involving the calculation of costs associated with the development and implementation of a mentoring program to train podiatrists to become endorsed prescribers. Costs were calculated using the Ingredients Method and examined from the perspective of a public health service provider, and the individual learner podiatrist. Breakeven analysis compared the cost of training a podiatry prescriber for endorsement against the potential benefit (savings) made by averting complications of an infected foot ulcer. A sensitivity analysis was conducted to allow for uncertainty in the results of an economic evaluation.

**Results:**

Total start-up cost for the podiatry prescriber mentoring program was $13, 251. The total cost to train one learner podiatrist was $30, 087, distributed between the hospital $17, 046 and the individual learner $13, 041. In the setting studied, a podiatry prescriber must avert 0.40 major amputations arising from an infected foot ulcer through prescribing to recover the cost of training. If in-kind training costs are included, total cost increases to $50, 654, and the breakeven point shifts to 0.68 major amputations averted.

**Conclusion:**

The economic benefits (savings) created by an endorsed prescribing podiatrist over their career in a public health service are likely to outweigh the costs to train a podiatrist to attain endorsement. Further research is required to help understand the effectiveness of podiatry prescribing in reducing diabetic foot related complications and the potential economic impact of podiatry prescribers on this health condition.

## Background

Non-medical prescribers have been practicing in Australia for over 10 years. These health professionals include nurse practitioners, optometrists, pharmacists and podiatrists [1]. The Australian Health Practitioner Regulation Agency (AHPRA) regulates each of these professions endorsed to prescribe [2]. All of these non-medical prescribers have a designated list of medications relevant to their job roles which they may utilise [3].

**Fig. 1:**
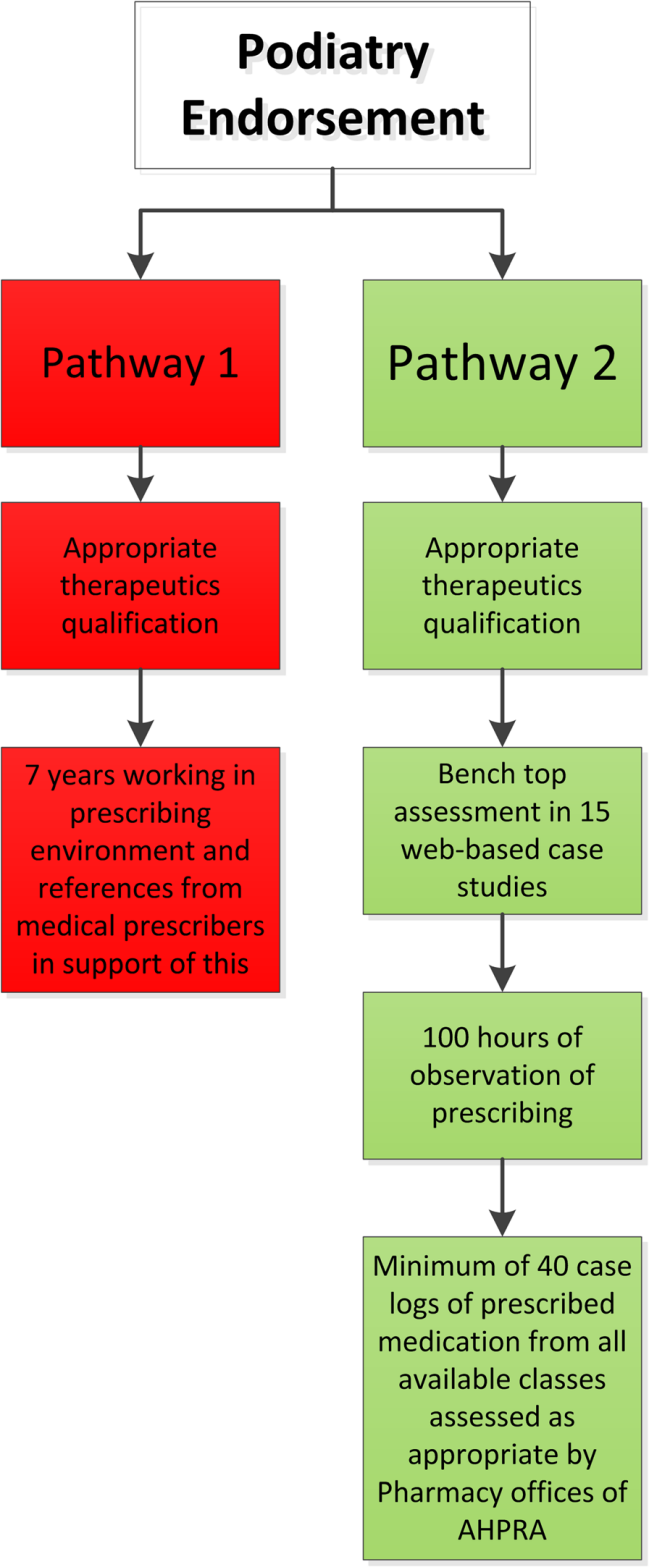
Current Pathways to Endorsement for Scheduled Medicines [5]

Within Australia, national legislation allows endorsed podiatry prescribers to prescribe and/or dispense to a limited amount of Schedule 2, 3, 4 and 8 medications [4]. Podiatrists may obtain endorsement to prescribe through either recognition of substantial exposure in a prescribing area; or through a university qualification with appropriate therapeutic subjects, 100 h of supervision practice and submission of 40 or more log cases demonstrating that the podiatrist has observed the prescription of all classes of drugs available to them (Fig. 1: Current endorsement process) [5]. Due to the time and supervision requirements to obtain endorsement (academically and clinically), the uptake of prescribing has been limited within the profession. Across both private and public sectors, there are currently only 86 (< 2%) endorsed prescribing podiatrists [6].

In the management of diabetes and high-risk patients, timely treatment with scheduled medicines is critical to prevent severe infections such as septicaemia, osteomyelitis and reduce the risk of lower extremity amputation [7]. More than 40% of diabetes related foot ulcers will become infected at some point, with more than half of those requiring hospitalisation, many will result in amputation [8, 9]. Hindered access to medical specialists can result in significant delays before these therapies can commence. These delays can result in increased severity of the presenting complaint or development of secondary complications. This is an area that a podiatrist may be best positioned to act and utilise their prescribing skills.

The primary aim of this study was to identify the costs associated with developing and implementing a podiatry prescribing mentoring program within a public health service. The secondary aim was to compare the cost of this program against potential healthcare savings produced by averting complications related to infected foot ulceration.

## Methods

### Study design

This was a cost-description analysis, defined as a calculation of costs but not associated benefits, and without comparison to another program [10]. Costs associated with the development and implementation of a mentoring program to train podiatrists to become endorsed prescribers were calculated. Peninsula Health (QA/18/PH/11) approved this research as being exempt from needed Human Research Ethics approval.

### Setting

**Fig. 2:**
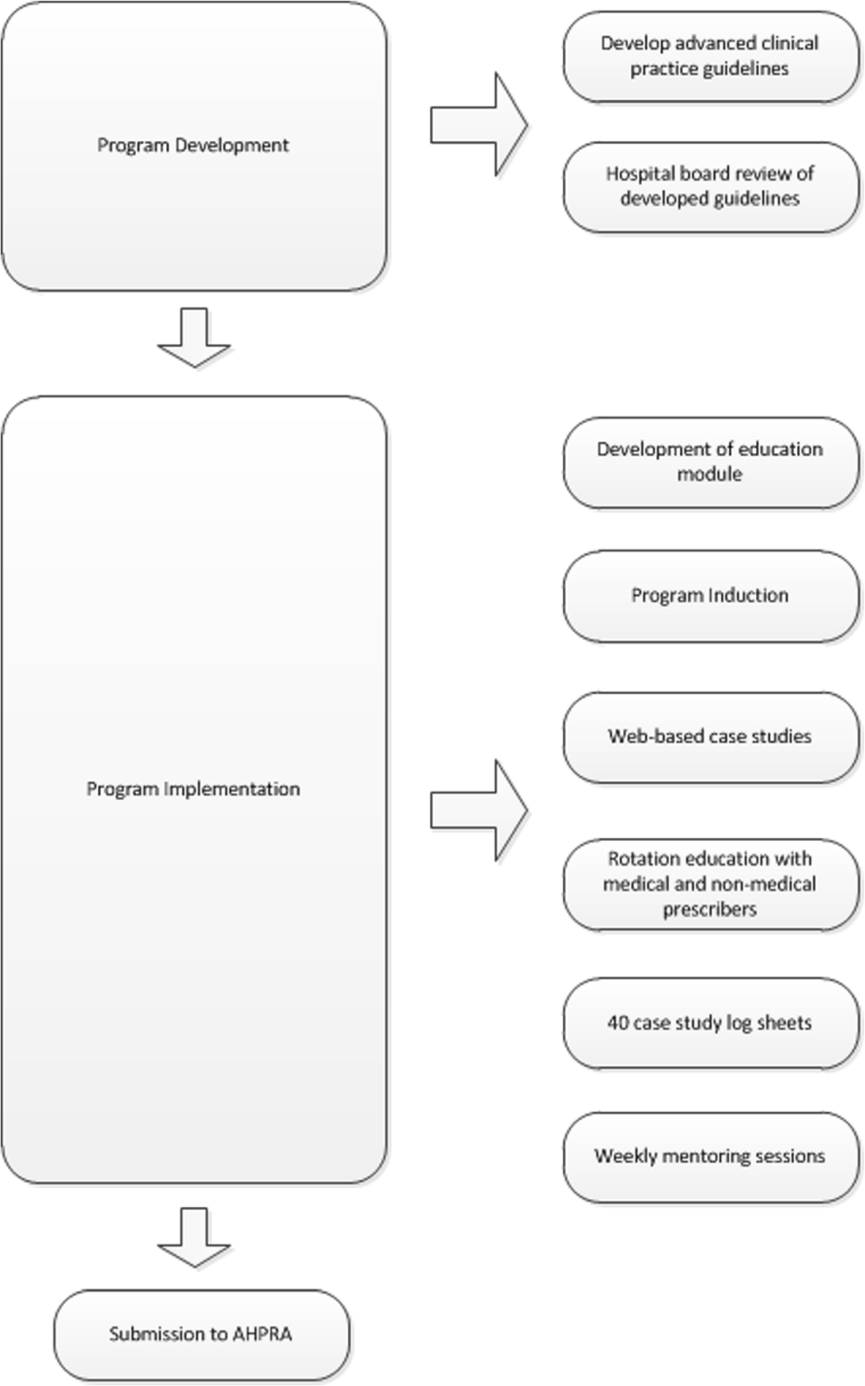
Overview of the mentoring program

Peninsula Health (public health service) developed an Endorsed Prescriber Podiatrists (S4 medications) mentoring program funded by the Department of Health and Human Services Victoria Advanced Practice Grant. In developing the mentoring program, locally required policies for podiatry prescribers in the public health system and a structure to mentor podiatrists while undertaking the requirements for prescribing endorsement (Pathway 2) were established. Peninsula Health is a tertiary teaching hospital, taking placement for undergraduate medicine, nursing and allied health students and hosts medical registrars for accredited training posts. Podiatrist undertook supervised practice by observing cases with medical students and registrars. These may have included outpatients appointment with a vascular surgeon for antibiotic prescription, in theatre with an orthopaedic surgeon to understand anaesthetic use and post-operative pain management or in an outpatient orthopaedic clinic for corticosteroid injection. This is provided to all medical students as part of their training with consultants and registrars, costing of their time was considered business as usual. The Peninsula Health model of mentoring and supervised practice was used as the basis for the present costing study, and therefore unless specified otherwise, 2017 Peninsula Health specific costs were used. The design of the mentoring program was based on the 2017 AHPRA requirements for Pathway 2 (Fig. 1). We considered activities involved with the development and implementation of the mentoring program (Fig. 2).

### Data collection and analysis

Costs were calculated using the Ingredients Method, a costing approach commonly used in educational economics research [11, 12]. Costs were examined from the perspective of the public health service (Peninsula Health) and the individual learner podiatrist. All values are reported in 2017 Australian Dollars. We expect that the typical learner would complete all activities within one year (the costing time horizon), and therefore no discounting was applied. The Ingredients Method was applied to the Podiatry Prescribing Program according to the following steps: 1. Identify and specify resource items, 2. Measure volume of resources in natural units, 3. Assign monetary prices to resource items, 4. Analyse and report costs [3]. Table 1 provides details of how each resource item cost was calculated.

**Table 1:** Description of cost calculation according to resource item

Resource Item	Description
Learner podiatrist paid time	This is time spent during work hours and is considered a cost to the hospital. Assumed to be a grade 2 year 4 podiatrist, with wage rate calculated according to the 2017 enterprise bargaining agreement [20]. On-costs of 25% are applied. Volume of time was calculated according to case study learning log documentation.
Learner podiatrist unpaid time	This is time spent outside of work hours and is considered a cost to the individual. Calculated at the marginal overtime rate of 50% of usual wage without on-costs [20]. Volume of time was estimated by learners.
Endorsed prescriber time	This is an individual who is endorsed to prescribe podiatric therapeutics, and is therefore eligible to supervise a learner. Mentoring occurs during work hours and is considered a cost to the hospital. Our calculations assume this is a grade 4 year 4 podiatrist [20]. On-costs of 25% are applied. Volume of time was calculated according to time-log documentation.
Administrator time	This individual works for the hospital and is involved with the development and organisation of the mentoring program, and has podiatry specific knowledge. We assume a grade 2 year 4 podiatrist [20]. On-costs of 25% are applied. Volume of time was calculated according to time-log documentation.
Senior pharmacist time	This is an experience pharmacist who is involved in leadership, supervision and education. Their role is to assist with the development of the program. Calculations assume a grade 3 year 4 pharmacist, with on-costs of 25% applied [23]. Volume of time spent on development was designated when the task was set. There is also ongoing pharmacist support for prescribing, however this is considered a normal function of their work and has not been calculated.
Committee member time	Various hospital committees reviewed the new clinical practice guidelines developed to support this program. For simplicity, we have assumed all individuals involved with these committees are at a similar level to the podiatry head of department, and assumed wage rates at grade 4 year 4 podiatrist level [20]. Total time taken was estimated according to the average number of agenda items and number of meeting attendees.
Librarian time	A medical librarian introduces the various learning resources available to learner podiatrists. Wage rate was calculated using the 2017 enterprise bargaining agreement [20]. This is a scheduled amount of time. Any ad-hoc support is considered a normal function of their work.

### Breakeven analysis

Breakeven analyses calculate the point at which the costs became equal to the benefits [12]. In this analysis, the cost of training a podiatry prescriber was compared against the potential benefit (savings) made by averting complications of an infected foot ulcer, considering the savings from averting ongoing conservative management, a minor amputation, and a major amputation. The diabetic ulcer treatment costing data were developed through a whole of podiatry service approach [13]. All audited health records included patients who presented between January and July 2015 at two health organisations in Melbourne, Australia, where an ulcer was coded as the primary reason for visit. Costing data accommodated:

- The number of consultations (from health records)
- Estimated podiatrist wage at 2016–2017 (including on costs) - $53 AU
- Length of stay (if an inpatient according to the National Weighted Activity Unit calculator and Diagnosis Related Group codes) [14, 15]
- Number and type of imaging related to the ulcer, number and type of microbiology taken for the ulcer. The value of microbiology and imaging costs were determined using the Australian Medicare fees as the weight. [16]
- Market costs of all consumables used within an appointment where sharps or low frequency ultrasonic debridement and wound dressing were undertaken. Consumable costs were developed whilst undertaking a randomised control trial run in parallel to this research within the same health service [14] - $24 AU (sharps only) or $86 AU (low frequency ultrasonic debridement with or without sharps).

Specifically, the saving was calculated as the difference between the healthcare cost of a resolved foot ulcer compared to the cost of the complication. This analysis excluded start-up costs, and did not apply discounting to future benefits for simplicity. The breakeven point provides an indication of how many patient events must be averted to recover the initial cost of training by the health service.

### Sensitivity analysis

A deterministic sensitivity analysis was conducted. The sensitivity analysis is the typical way uncertainty is characterised in economic studies [10], with the deterministic approach most appropriate due to the absence of stochastic data. In this approach, the robustness of the results are tested under a range of selected conditions. Table 2 describes the tested scenarios.

**Table 2:** Description of sensitivity analysis scenarios

Scenario	Description
1) Includes hospital cost for rotation education	Estimates the cost to the hospital of conducting rotation education according to the average cost of medicine trainees of $4376/month with 40 h weeks [24]
2) Uses house medical officer as endorsed prescriber	Estimates the program cost if the endorsed prescriber role is filled by a year 3 House Medical Officer at an hourly rate of $38.66, with a 4% pay increase to 2017 values, and 25% on-costs [25]
3) Uses medical officer as endorsed prescriber	Estimates the program cost if the endorsed prescriber role is filled by a year 5 Medical Officer at an hourly rate of $58.99, with a 4% pay increase to 2017 values, and 25% on-costs [25].
4) Graduated from a recognised program in the last 7 years	AHPRA require podiatrists eligible to apply for endorsement to have completed a recognised therapeutics program in the last 7 years. Those who satisfy this requirement do not have to do an additional podiatric therapeutics course, therefore creating a cost saving.
5) Increase 20%	Increases all implementation costs by 20%.
6) Decrease 20%	Decreases all implementation costs by 20%.

## Results

### Program costs

The total start-up cost for the podiatry prescriber mentoring program was $13,251 (Table 3). This includes development of hospital based practice guidelines, review of developed guidelines within established health care infrastructure and development of supporting learning resources.

**Table 3:** Program start-up costs

Activity	Resource item	Volume	Price	Cost
Development of advanced clinical practice guidelines	Administrator time	90 h	$56.40/h	$5076
	Endorsed prescriber time	20 h	$76.33/h	$1527
	Senior pharmacist time	4 h	$69.10/h	$276
Hospital review of developed guidelines	Committee member time	2 h	$76.33/h	$153
Development of learning resources	Senior pharmacist time	90 h	$69.10/h	$6219
			TOTAL	$13,251

The total cost of implementing the program to train one learner podiatrist was $30,087. This cost was distributed between the hospital $17,046 (57%) (Table 4) and the individual learner $13,041 (43%) (Table 5). The largest cost incurred by the hospital was that of the learner podiatrists’ time while they were undertaking educational rotations with colleagues of different professional (medical) specialisations, to build their 40 assessable case log reports. It is important to recognise that the more senior the learner podiatrist, the higher their wage, and therefore the higher the cost associated with their learning. The learner podiatrists’ unpaid time in preparing their log sheets was the most significant contributor to the learner costs.

**Table 4:** Hospital costs to train one podiatry prescriber

Activity	Resource item	Volume	Price	Cost
Program induction	Endorsed prescriber time	1 h	$76.33/h	$76
	Learner podiatrist paid time	4 h	$56.40/h	$226
	Librarian time	1 h	$67.48/h	$67
Rotation education	Learner podiatrist paid time	188 h	$56.40/h	$10,604
Log sheet mentoring	Endorsed prescriber time	30 h	$76.33/h	$2290
	Learner podiatrist paid time	10 h	$56.40/h	$564
Post-mentoring activities	Endorsed prescriber time	20 h	$76.33/h	$1527
	Learner podiatrist paid time	20 h	$56.40/h	$1128
Program administration	Administrator time	10 h	$56.40/h	$564
			TOTAL	$17,046

**Table 5:** Learner costs to become a podiatry prescriber

Activity	Resource item	Volume	Price	Cost
Approved podiatric therapeutics program of study^a^	Course fee	1	$4875.03^b^	$4875
	Learner podiatrist unpaid time	260	$22.56 /hour	$5866
Web based case studies	Course fee	1	$250.00	$250
	Learner podiatrist unpaid time	2.5 h	$22.56 /hour	$56
Log sheet preparation	Learner podiatrist unpaid time	80 h	$22.56 /hour	$1805
AHPRA submission	Application fee	1	$189.00	$189
			TOTAL	$13,041

^a^Only required if learner has not completed an approved program of study within the last 7 years

^b^Based on University of South Australia Advanced Pharmacology for Podiatrists 2017 course fees

### Breakeven analysis

The breakeven analyses considered averting further complications of an infected foot ulcer, which would have otherwise gone on to have various outcomes ranging from scenario A to C (Table 6). Ongoing conservative management (scenario A) was the least costly and major amputation (scenario C) was the most costly. The breakeven point was analysed from a perspective of all costs (hospital and learner), and hospital only costs.

**Table 6:** Breakeven analysis

Infected foot ulcer outcome averted	Cost avoided	Overall breakeven	Hospital breakeven
A: Requires ongoing management (e.g. wound debridement, wound dressing, antibiotics)	$3948	7.62	4.32
B: Minor amputation: toe	$20,240	1.49	0.84
C: Major amputation: below knee or trans-metatarsal	$74,944	0.40	0.23

### Sensitivity analysis

The sensitivity analysis was carried out according to the scenarios listed in Table 2, and reported in Table 7. The sensitivity analysis considered the impact of each scenario (numbered 1 to 6) on implementation cost and breakeven point. For example, from scenario 1, by including an estimate of the cost to provide rotation education, the total cost to produce one podiatry prescriber increases by approximately $20,000, a cost born by the hospital. In order to recover this training cost, the podiatry prescriber needs to avert 0.68 potential major amputations, resolved after treatment by the podiatrist that included the use of prescribed medicines (scenario A), rather than the 0.40 needed to be averted in the original scenario where rotation education is contributed in-kind.

**Table 7:** Sensitivity analysis

Scenario	Total implementation cost	Hospital cost	Learner cost	Overall breakeven^a^
Original	$30,087	$17,046	$13,041	0.40
1) Includes rotation education cost	$50,654	$37,613	$13,041	0.68
2) Hospital medical officer as endorsed prescriber	$28,757	$15,716	$13,041	0.38
3) Medical officer as endorsed prescriber	$30,105	$17,064	$13,041	0.40
4) Recent podiatry graduate from recognised program	$19,346	$17,046	$2300	0.26
5) Increase 20%	$36,104	$20,455	$15,649	0.48
6) Decrease 20%	$24,070	$13,637	$10,433	0.32

^a^Using breakeven scenario C: major amputation avoided

## Discussion

The cost-description results show that the start-up costs compared to the implementation costs are relatively small within public health podiatry departments within Australia. The start-up cost relating to the content (i.e. the clinical practice guidelines and teaching materials) and costs related to tailoring the content/program to a particular hospital may be considered as business as usual or generic across health services [17]. Content, once developed, would ideally be widely transferable to other hospitals at no or minimal extra cost. Additional infrastructure or departmental costs are also associated with implementing a podiatry prescribing mentoring program within a health service. These costs should only be attributed to the learner podiatrists’ time while undertaking educational rotations to build their case logs.

Economic decision-making on program implementation cost to the hospital (and therefore the public health system) are important, particularly in an economic climate of increased budgetary constraints [18]. Given that cause and effect of educational interventions are difficult to isolate in the complex healthcare environment, comparing the breakeven point of an intervention against its estimated effect can enhance decision-making [19]. Given the relatively small hospital breakeven values of calculated, across the life of the prescribing podiatrist, it is likely that, within the Australian healthcare setting, an endorsed prescribing podiatrist will result in a net economic benefit.

In contrast, the return-on-investment for the learner podiatrist was less clear. Under the current enterprise bargaining agreement, endorsed prescriber podiatrists do not receive different wages to non-prescribers [20]. This is particularly relevant for those who have not completed an appropriate therapeutic program of study within the last 7 years, and must therefore meet additional training requirements.

Endorsed prescribing may also create intangible benefits to the hospital and podiatrist. Other professions have noted the advancement of skills, increases in job satisfaction, and retainment of staff [21]. The endorsed skill set broadens the podiatrists’ scope of practice, providing them with an extra clinical tool. An endorsed prescribing podiatrist may also be more employable or have increased opportunities for job promotion.

There are a number of limitations to consider when interpreting the study findings. The analysis did not consider the ongoing costs and benefits of using a podiatry prescriber compared to the standard usage of a medical prescriber. Additionally, the costs of providing wound care may differ between Australian states and over time. There is no in depth costings for outpatient podiatry services for wound care where amputation is not the outcome. Additionally, the costing model was undertaken in 2016–2017, since this time, consumables, podiatry wages, Medicare and hospital costs will all have been adjusted. Therefore, these costs may vary according to setting, podiatrist wages over time, and may only be applied to Australia. A podiatry prescriber may create a small comparative saving given that podiatry wages are generally lower than medical wages. However, this must also be balanced against administration time in prescribing. This administration time may be the amount of time taken to write the prescription given there is no template prescription pads for podiatry. It may also take into account the costs in identifying the correct prescription, such as time spent consulting with pharmacy or online prescribing referencing materials such as the Therapeutic Guidelines. Alternatively, there may be potential for differences in prescriber group effectiveness that also affects administration time in prescribing. There is currently insufficient evidence to determine whether there is any difference in the number of adverse events or resources use between medical and non-medical prescribers, although effectiveness has been found to be similar across a range of settings [22]. Rather than a direct economic benefit, one benefit from using a podiatry prescriber instead of a medical prescriber is to free up medical prescriber time for other tasks, allowing both professions to work at the full extent of their capacities, increasing overall system efficiency.

Learner costs should be interpreted with caution, as the amount of time spent preparing logs is likely to vary greatly from individual to individual. Additionally, individuals have different values for their time. These values depend on what the learner would have otherwise been doing, and the value they place on these alternatives. Further research is required to understand the economic and non-economic factors that may influence podiatrists’ decision to become an endorsed prescriber.

Further research is required to help understand the effectiveness of podiatry prescribing in reducing diabetic foot related complications and the potential economic impact of podiatry prescribers on this health condition. There are additional costs, not considered within the primary analysis. The largest of these is the cost of offering education with various specialised medical teams across the health service. The education occurs as part of business as usual for medical students, junior doctors or registrars, and cannot be accurately differentiated to be costed within the scope of this study. This cost has been treated as ‘good-will’ on behalf of the medical doctors who support the education process, as they would normally do so for medical learners. Other less significant costs include generic library resources, office supplies, and utilities, all of which are estimated as too small to effect the overall analysis.

## Conclusion

The results support the economic viability of training podiatrists to attain endorsement from the perspective of the health service. This is identified through the relatively small training cost compared to the potential savings achieved through averting future cost-consequences of clinical complications. It is crucial that appropriate practice guidelines are developed within established health care infrastructure that support a podiatrist to prescribe in the health service.

## Abbreviation

AHPRA: Australian Health Practitioner Regulation Agency
